# Profils cliniques, biologiques et étiologiques des ostéomalacies Clinical, biological and etiological features of osteomalacia

**DOI:** 10.11604/pamj.2020.37.215.26407

**Published:** 2020-11-03

**Authors:** Dhia Kaffel, Kaouther Maatallah, Hanene Lassoued Ferjani, Wafa Triki, Dorra Zarati, Wafa Hamdi

**Affiliations:** 1 Service de Rhumatologie, Institut KASSAB, Manouba, Tunisie

**Keywords:** Ostéomalacie, vitamine D, apport calcique

## Abstract

**Introduction:**

l´ostéomalacie est une ostéopathie raréfiante secondaire à un défaut de minéralisation de la trame osseuse. Son diagnostic est le plus souvent porté au stade de complications car ses manifestations sont diverses et souvent méconnues. But: étudier les profils cliniques, biologiques et étiologiques de l´ostéomalacie.

**Méthodes:**

il s´agit d´une étude rétrospective colligeant tous les dossiers d´ostéomalacie hospitalisés entre Mai 2006 et Janvier 2014.

**Résultats:**

notre étude a inclus 30 cas d´ostéomalacie avec un âge moyen de 55 ans [29 ans - 82 ans]. Une nette prédominance féminine était notée avec un sexe ratio de 0.11. Tous nos patients avaient un régime hypo-calcique et une seule patiente avait un régime suffisant en vitamine D. Tous les patients présentaient des douleurs osseuses à l´examen et 80% d´entre eux présentaient un trouble de la marche. Les déformations ont été notées dans la moitié des cas. Sur le plan biologique, la baisse de la 25 OH vit D était constamment retrouvée suivie par l´augmentation des phosphatases alcalines (90%), alors que l´hypocalcémie et l´hypophosphorémie étaient présentes chez respectivement 46,6 et 50% des cas. La carence en vitamine D était la cause retenue dans la majorité des cas (86.6%). Une malabsorption a été notée dans 2 cas.

**Conclusion:**

à travers notre travail, nous avons mis en évidence plusieurs formes évoluées d´OM. Ceci impose un dépistage précoce et une enquête étiologique minutieuse.

## Introduction

L´ostéomalacie (OM) est une ostéopathie métabolique raréfiante diffuse de l´adulte caractérisée par un défaut de minéralisation de la matrice protéique osseuse. Les causes de l´OM sont variées et le plus souvent en rapport avec un déficit sévère et prolongé en vitamine D. L´OM se manifeste essentiellement par des douleurs osseuses associées à une myopathie proximale, mais elle peut se révéler par plusieurs symptômes non spécifiques. La biologie varie en fonction de l´étiologie, néanmoins l´augmentation des phosphatases alcalines ainsi que la baisse de la Vit D sont les éléments les plus fréquemment retrouvés. De ce fait, les manifestations clinico-biologiques de l´OM sont diverses et souvent méconnues et le diagnostic est le plus souvent porté à un stade tardif. L´objectif de notre travail est d´étudier les profils cliniques, biologiques et étiologiques de l´OM.

## Méthodes

Il s´agit d´une étude rétrospective portant sur tous les dossiers d´OM colligés dans notre service sur une période de 7 ans et 8 mois allant de Mai 2006 jusqu´à Janvier 2014. Pour chaque dossier, nous avons recueilli un ensemble de données en remplissant une fiche préconçue. Pour les données manquantes, nous avons contacté par téléphone, lorsque c´était possible, les patients. Nous avons recueilli les données socio démographiques ainsi que les données cliniques, et biologiques. L´apport calcique a été calculé par le questionnaire fréquentiel de Fardellone [1]. L´apport en vit D a été évalué en se référant au répertoire général des aliments [2]. L'analyse statistique a été réalisée à l´aide du logiciel Statistical Package for Social Sciences (SPSS) for Windows version 15.0. Le test du Chi 2 de Pearson a été utilisé pour comparer des pourcentages sur séries indépendantes et en cas de sa non-validité, on a eu recours au test exact bilatéral de Fisher. La comparaison de deux moyennes a été effectuée par le test t de Student sur séries indépendantes. Dans tous les tests statistiques, le seuil de signification a été fixé à 0,05.

## Résultats

Données épidémiologiques: l´âge de nos patients variait de 29 à 82 ans avec une médiane de 60 ans et une moyenne à 55ans. Dans notre série une prédominance féminine était notée: 27 femmes et 3 hommes avec un sexe ratio (H/F) de 0.11. Une patiente était d´origine mauritanienne et le reste d´origine tunisienne répartis comme suit: 75% du Nord, 10% du centre et 10% du sud. Le milieu socioéconomique était précisé chez 22 malades (73%): 18 vivaient dans un milieu urbain alors que 4 vivaient dans un milieu rural. Dans notre série 71% des femmes étaient voilées. Une seule patiente était de race noire, les autres patients avaient un teint blanc ou métis. Dix-huit malades avaient des antécédents pouvant interférer sur le métabolisme osseux (Tableau 1). Tous nos patients avaient un régime hypo-calcique. L´apport calcique variait entre 213 et 691 mg/j avec une moyenne de 416 mg/j et une médiane de 411 mg/j. Dans notre série, l´apport en VIT D était compris entre 0.5 et 5.2 µg/j avec une moyenne à 2.87 µg/j. Une seule patiente avait un apport suffisant en Vit D.

**Tableau 1:** répartition des antécédents pouvant interférer sur le métabolisme osseux

Antécédents	Nombre des cas
Gastrectomie subtotale	1
Insuffisance rénale chronique	2
Spondyloarthrite	4
Anémie de Biermer	1
Polyarthrite rhumatoÏde	1
Insuffisance hypophysaire congénitale	1
Néphrectomie unilatérale	1
Alitement	7
Fracture	10

Données cliniques: les circonstances de découverte de l´OM étaient cliniques chez tous les patients et étaient prédominées par les douleurs ostéo-articulaires, les troubles de la marche et l´impotence fonctionnelle. Tous les patients se plaignaient de douleurs pelviennes. Trente-sept pour cent des patients avaient des myalgies diffuses et seulement 3% se plaignaient de crampes au niveau des membres inférieurs. Dix-sept malades (56.6%) représentaient une impotence fonctionnelle. Elle était localisée au niveau des deux membres inférieurs chez 15 d´entre eux. Quatre-vingts trois pour cent des patients avaient un périmètre de marche diminué et 4 d´entre eux avaient besoin de chaise roulante pour le déplacement. Enfin, un patient présentait par ailleurs une claudication médullaire secondaire à un tassement vertébral dorsal. Les troubles de la marche étaient retrouvés chez 25 malades (marche dandinante (62.5%), marche à petit pas (21%) et marche impossible (16.5%)). Les déformations osseuses étaient constatées chez 50% des malades (Tableau 2). Les douleurs osseuses aggravées par la palpation étaient retrouvées à l´examen de tous les malades. Seize patients (53%) avaient une mobilité articulaire diminuée prédominante au niveau des hanches (9 cas). Un raccourcissement du membre inférieur était retrouvé à l´examen d´un patient qui présentait une fracture du col fémoral. Les myalgies étaient présentes chez 13 patients. Une amyotrophie était retrouvée chez 3 patients (10%) et le signe du tabouret était positif dans 60% des cas. Des signes neurologiques étaient retrouvés à type de reflexe ostéo-tendineux abolis (2 patients), d´hypoesthésie au niveau des membres inférieurs (2 patients), de paraparésie avec un syndrome cordonal postérieur (1 patiente avec une anémie de biermer).

**Tableau 2:** les déformations osseuses à l'examen clinique

Déformation	Nombre de malades
Thorax en entonnoir	3
Saillie du sternum	2
Cyphose dorsale	6
Scoliose lombaire	4
Effacement de la lordose lombaire	5
Hyperlordose lombaire	3
Genu Varum	3
Membres inférieurs en parenthèse	1
Flessum hanche G	1

**Figure 1:**
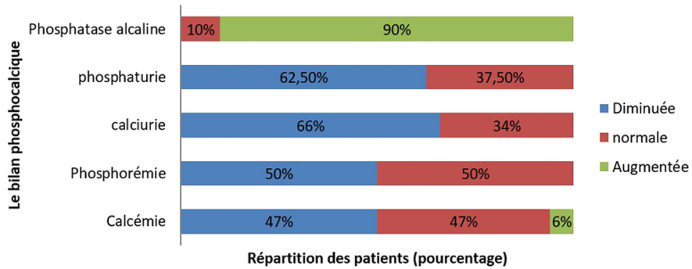
les résultats du bilan phosphocalcique

Les données biologiques: la calcémie variait dans notre série entre 1.6 et 2.95 mmol/l avec une moyenne de 2.26 mmol/l. La calcémie était abaissée chez 46,6% des patients. Elle était normale chez 46,6% d´entre eux alors qu´elle n´était augmentée que chez 6,6% des cas. La phosphorémie variait chez nos patients entre 0.1 et 1.35 mmol/l avec une moyenne de 0.78. Elle était abaissée chez la moitié des patients et normale pour le reste. La calciurie moyenne était de 1.92 mmol/24h (extrêmes: 0.12- 6.5 mmol/24h) et elle était abaissée chez les deux tiers des patients. La phosphaturie moyenne était de 13.46 mmol/24h [2.5- 6.8 mmol/24h] et L´hypophosphorémie était notée dans 62.5% des cas. Les valeurs des phosphatases alcalines étaient comprises entre 68 et 2516UI/l avec une moyenne de 651UI/l: Celles-ci étaient élevées chez 27 patients (elles étaient normales dans les cas restants) (Figure 1). La créatinémie était élevée chez un patient insuffisant rénal. Une hypo albuminémie était observée chez 40% des patients et elle était normale pour le reste. Le dosage de la PTH a été réalisé chez 22 patients. Quatorze malades (63.6%) avaient des taux élevés. On n´a pas noté de valeurs abaissées de la PTH. Concernant la 25-OH-vitD, son dosage a été réalisé chez tous les malades. Tous les patients avec une OM carentielle avaient une valeur abaissée avec une moyenne de 6,25ng/ml. Un bilan inflammatoire était fait chez tous les patients parmi lesquels 2 avaient un syndrome inflammatoire biologique et une anémie était observée chez 4 patients. Une carence en vitamine B9 et B12 était observée chez une patiente atteinte d´une anémie de Biermer. La sérologie de la maladie cœliaque était pratiquée chez 2 patients et était positive dans un cas. Enfin, la chromatographie des acides aminés urinaires a relevé une hyperaminoacidurie axée sur la glycine et l´alanine chez un patient souffrant d´un syndrome de Fanconi incomplet.

### Les étiologies

L´OM par déficit en vit D était retenue chez 26 patients (86.6%): (24 patients souffraient d´une carence d´apport en vit D et 2 patients avaient une malabsorption digestive secondaire à une résection chirurgicale gastrique et à une maladie cœliaque). L´OM hypophosphorémique était retenue chez 3 patients dont un patient avec un syndrome de Fanconi incomplet et un autre avec une OM oncogénique causée par une tumeur de la fosse nasale. Une OM par insuffisance rénale était retrouvée chez 1 seul patient (3.3%).

Etude analytique: les différentes manifestations cliniques ont été comparées entre les patients par rapport à la médiane d´âge (60 ans), au sexe et aux étiologies (Tableau 3). Nous avons trouvé que les patients qui présentaient des troubles de la marche étaient plus âgés (53,24 ± 14,5) que ceux qui n´en présentaient pas (70 ± 7,1) de façon significative (p=0,019). Les paramètres biologiques ont été comparés selon l´étiologie de l´OM. Nous avons observé une hypovitaminose D en cas d´étiologie carentielle de façon significative (p=0,002).

**Tableau 3:** présence des manifestations cliniques selon l'âge, le sexe et l'étiologie

Manifestations cliniques	Age˂60 ans	Age≥60 ans	P	Sexe masculin	Sexe féminin	p	Etiologies carentielle	Etiologies non carentielle	p
Impotence fonctionnelle	9	8	0,638	3	14	0,167	14	3	0,633
Troubles de la marche	5	20	0,019	3	22	0,567	20	5	0,373
Myalgies	8	5	0,525	2	11	0,397	10	3	0,367

## Discussion

L´OM est une pathologie rare ce qui explique le nombre restreint de malades dans les différentes séries de la littérature. Elle prédomine chez la femme, ce qui concorde avec notre étude. Cette prédominance féminine peut être expliquée par la prépondérance de l´hypovitaminose D chez les femmes (habitudes vestimentaires, grossesse, allaitement). L´OM peut être révélée par plusieurs symptômes non spécifiques et le diagnostic peut passer inaperçu dans les premiers stades de la maladie [3]. Dans notre étude, ainsi que dans plusieurs séries de la littérature, la principale circonstance de découverte était les douleurs ostéo-articulaires (Basha [4] et Bingham [5]: 94% des cas, Gifre [6]: 89% des cas et Aissa [7]: 73% des cas). Les résultats des examens biologiques dépendent de l´étiologie de l´OM [8]. Une hypocalcémie est surtout retrouvée dans les OM carentielles [9]. Dans notre série la calcémie était diminuée dans 46,6% des cas. Cette fréquence se rapproche de celles rapportées par plusieurs auteurs (Bingham [5]: 47%, Reginato [10]: 46%, Triki [11]: 65%, Gifre [6]: 37%, Adrar [12]: 55%). Différentes études ont été réalisées pour mieux étudier la sensibilité de l´hypocalcémie et sa valeur prédictive. Ainsi, Peach [13] a réalisé 2 études sur une population asiatique installée en grande Bretagne. La première étude était rétrospective et faite d´une part sur 50 patients ayant une OM histologique et d´autre part sur 50 autres témoins hospitalisés pour un problème chirurgical et ayant bénéficié d´une biopsie osseuse. La deuxième étude était prospective et réalisée sur 62 patients hospitalisés chez qui l´OM histologique était retrouvée dans la moitié des cas. Ces 2 études ont révélé des taux de faux négatifs (calcémie normale et biopsie négative) chiffrés respectivement à 38 et 48%, alors que les taux de faux positifs (hypocalcémie avec biopsie négative) étaient bas et chiffrés à 8 et 6,5%. Adrar [12] et Campbell [14] rapportent aussi dans leurs études des taux de faux négatifs élevés à 45 et 90% respectivement. Ces différents résultats expliquent pourquoi beaucoup d´auteurs considèrent la valeur de la calcémie comme un test peu sensible pour le diagnostic de l´OM.

L´hypophosphorémie est retrouvée dans 30 à 70% des séries de la littérature. Dans notre série, ainsi que celle de Bingham [5] et celle de Triki [11] l´hypophosphorémie était retrouvée dans la moitié des cas. Peach [13] rapporte que la phosphorémie seule a une faible valeur prédictive dans le diagnostic d´OM. En effet, dans ses deux études rétrospective et prospective, le taux de faux négatif était élevé à 81 et 96% respectivement. Au cours de l´OM, l´activité des phosphatases alcalines est habituellement augmentée. En effet, selon les séries de la littérature, l´hyperphosphatasémie est retrouvée dans 73 à 100% des cas, ce qui est le cas de notre série. Selon certaines études, l´hyperphosphatasémie a la meilleure valeur prédictive pour le diagnostic de l´OM. Ceci a été démontré par Peach [13] qui, dans son étude rétrospective, a rapporté des taux bas de faux négatifs (14%) et des faux positifs (8%). En cas de déficit en vit D, l´hypocalciurie est généralement un signe précoce. Selon Bingham [5], l´excrétion urinaire de calcium chez les patients ayant une OM histologiquement confirmée, avait une faible sensibilité. En effet, il n´a trouvé l´hypocalciurie que chez 18% des patients. Mais, selon d´autres auteurs (Bregeon [15] et Frame [16]), l´hypocalciurie apparait comme un signe fidèle de l´OM malgré qu´elle ne soit pas incluse dans les scores de diagnostic d´OM. Dans les OM carentielles, la diminution du taux de 25-hydroxyvitamine D est constamment retrouvée alors que le taux de 1,25 hydroxyvitamine D (calcitriol) peut être diminué ou normal selon les séries. Ceci s´explique par le fait que la 1,25 hydroxyvitamine D présente une variation nycthémérale [17]. Cependant, l´interprétation de la baisse de la 25OH vit D doit être prudente. En effet, Sebert [18] a retrouvé que 55% des patients âgés entre 65 et 97ans avaient un taux de 25-OH-vit D inférieur à 15 ng /ml. L´hypocalcémie et le déficit en vit D peuvent provoquer une hyperparathyroïdie secondaire avec augmentation de la sécrétion de la PTH. Cette hyperparathyroïdie secondaire est responsable d´une augmentation de la réabsorption tubulaire de calcium et d´une diminution de celle du phosphore ce qui aggrave l´hypophosporémie. Elle stimule en plus la résorption ostéoclastique [19].

Dans une étude tunisienne (Triki *et al*.) [11] en 2007 incluant 20 cas, les étiologies retrouvées étaient dominées comme dans notre série par les OM carentielles (80% des cas avec un cas de maladie cœliaque). Ils avaient aussi noté 2 cas d´insuffisance rénale chronique, 1 cas de diabète phosphoré et 1 cas de cirrhose. De même, dans l´étude d´Aissa *et al*. [7] portant sur 33 patients, une OM par carence d´apport était retrouvée dans la majorité des cas (45,5%). Une OM par malabsorption intestinale était rapportée plus fréquemment que dans les autres séries de la littérature (27,3% des patients: 7 cas de maladie cœliaque, 1 cas de cirrhose biliaire primitive et 1 cas de Gastrectomie). Les autres étiologies étaient une insuffisance rénale chronique chez 15,1% des patients et une fuite rénale de Phosphore chez 12,1% des patients (dont 1cas de syndrome de Fanconi, 1 cas d´OM oncogénique en rapport avec une tumeur mésenchymateuse vésicale et 2 cas d´acidose tubulaire distale dont un était secondaire à un syndrome de Gougerot Sjögren). Dans la série de Fruigui [20] incluant 16 cas d´OM, en dehors de l´insuffisance rénale chronique, les étiologies déterminées dans la moitié des cas étaient: 2 cas de syndrome de Gougerot-Sjögren, 2 cas de maladie coeliaque, 1 cas de syndrome de Fanconi (secondaire à un myélome multiple), 1 cas d´hypophosphorémie Familiale et 1 cas d´acidose tubulaire proximale. Par contre, dans une étude Espagnole, Gifre *et al*. [6], avaient rapporté 28 cas d´OM. L´origine carentielle était moins fréquente (46% des cas) avec un cas de chirurgie bariatrique et un cas d´OM en rapport avec la prise d´antiépileptique. L´OM hypophosphorémiante était l´étiologie la plus fréquemment retenue (50% des patients): origine héréditaire dans 6 cas, une fuite urinaire de calcium dans 2 cas, une tubulopathie dans un cas et un cas d´OM oncogénique. Enfin l´hypophosphatasie était retrouvée chez un patient. Dans l´étude de Peach *et al*. [13], 50 cas d´OM ont été rapportés. Les étiologies retrouvées étaient un déficit en vit D dans 40% des cas, un trouble du métabolisme du phosphore dans 4% des cas et une malabsorption dans 18% des cas répartis comme suit: 5 cas de résection intestinale, 3 cas de chirurgie by pass jéjunal et un cas de maladie de Crohn. Ainsi, dans notre étude, l´étiologie prédominante, comme dans la majorité des séries de la littérature, était l´OM carentielle.

## Conclusion

A travers notre travail, nous avons mis en évidence plusieurs formes évoluées d´OM. Ceci impose un dépistage précoce et une enquête étiologique minutieuse. D´autres études multicentriques permettront de mieux évaluer cette maladie.

### Etat des connaissances sur le sujet

- L´ostéomalacie est une ostéopathie métabolique raréfiante diffuse de l´adulte caractérisée par un défaut de minéralisation de la matrice protéique osseuse;
- Les manifestations clinico-biologiques de l´ostéomalacie sont diverses et souvent méconnues;
- Le diagnostic est le plus souvent porté à un stade tardif.

### Contribution de notre étude à la connaissance

- Dans notre étude, la principale circonstance de découverte était les douleurs ostéo-articulaires;
- La biologie varie en fonction de l´étiologie, néanmoins l´augmentation des phosphatases alcalines ainsi que la baisse de la Vit D sont les éléments les plus fréquemment retrouvés;
- Nous avons mis en évidence plusieurs formes évoluées d´ostéomalacie. Ceci impose un dépistage précoce et une enquête étiologique minutieuse.
